# Prenatal Lethal Diagnosis of 8p23.1 Duplication Syndrome Associated with Omphalocele and Encephalocele

**DOI:** 10.1155/2023/5958223

**Published:** 2023-02-25

**Authors:** Melissa A. Hicks, Salah Ebrahim, Bernard Gonik

**Affiliations:** ^1^Department of Pathology, Detroit Medical Center University Laboratories, Detroit, MI, USA; ^2^Department of Cytogenetics, Spectrum Health, Grand Rapids, MI, USA; ^3^Department of Obstetrics and Gynecology, Wayne State University, Detroit, MI, USA

## Abstract

Despite increased prenatal and postnatal use of array comparative genomic hybridization (aCGH), isolated 8p23.1 duplication remains rare and has been associated with a widely variable phenotype. Here, we report an isolated 8p23.1 duplication in a fetus with an omphalocele and encephalocele that were incompatible with life. Prenatal aCGH demonstrated a 3.75 Mb de novo duplication of 8p23.1. This region encompassed 54 genes, 21 of which are described in OMIM, including *SOX7* and *GATA4.* The summarized case demonstrates phenotypic features not previously described in 8p23.1 duplication syndrome and is reported in order to enhance understanding of the phenotypic variation.

## 1. Introduction

The use of microarray technologies has expanded the potential for prenatal diagnosis. However, due to its ability to sensitively detect many novel abnormalities, it is difficult to predict the associated phenotype. Patient counseling regarding the genetic findings is thus challenging for clinicians. Isolated duplication of 8p23.1 has been described in several case reports, with a continuum of the fragility of the 8p chromosomal region having been observed [1–5]. The population prevalence of the 8p23.1 duplication syndrome (8p23.1 DS) has been estimated to be about 1 in 58,000 [2]. Variable phenotypes have been described, though duplication of 8p23.1 appears to be associated most with developmental delay, dysmorphism, macrocephaly, and otitis media [2]. Here, we describe a patient with isolated duplication of 8p23.1 diagnosed prenatally using array comparative genomic hybridization (aCGH) and summarize phenotypic findings not previously reported.

## 2. Clinical Report

A multigravid patient in her mid-30s was referred for maternal-fetal medicine consultation due to sonographic findings of omphalocele and encephalocele. The patient was in a nonconsanguineous relationship. Her medical history was notable for hypertension, which did not require medication. There was no history of congenital anomalies or developmental delays in previous children.

During the fetal anatomic survey, a large omphalocele was noted, containing both liver and bowel. A posterior encephalocele was also visualized, with a flattened facial profile. The patient underwent cell-free DNA screening earlier in the pregnancy which estimated a low risk (<1/10,000) for trisomies 13, 18, 21 and monosomy X, and a decreased risk for triploidy and 22q11.2 deletion syndrome (1/2,900). Carrier screening for 27 autosomal recessive and X-linked conditions, including Smith–Lemli–Opitz syndrome, was noncontributory. She was offered an amniocentesis for genetic studies aimed at diagnosing the cause of these ultrasound findings. The patient consented and underwent amniocentesis at 23 weeks' gestation.

**Figure 1:**
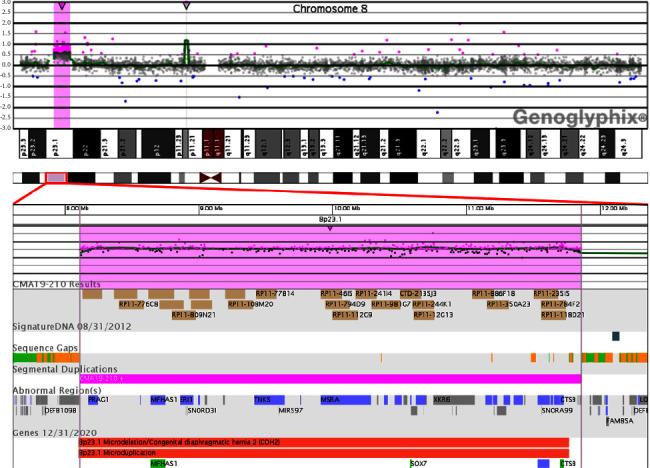
Analysis of 8p23.1 region. The aCGH oligonucleotide data plot for chromosome 8 depicts a 3.75 Mb microduplication (shaded in pink) of the 8p23.1 region including the *SOX7* gene.

Genomic microarray using the oligonucleotide-single nucleotide polymorphism whole genome Agilent GGXChip + SNP version 1.0 (Signature Genomics Inc.) was performed on fetal DNA isolated from direct amniotic fluid. The results demonstrated a 3.75 Mb interstitial microduplication (copy number gain) of the short arm of chromosome 8 at 8p23.1 (arr[GRCh37] 8p23.1(8108992–11858460)x3). The duplicated region contained 54 genes: 21 OMIM genes and 33 others per ClinGen [6], including the *SOX7* and *GATA4* genes (Figure 1). No other clinically significant copy number variants were noted. The duplication was not visible on fetal karyotype (46, XX).

Due to the fetal microduplication, focused microarray analysis of the 8p region was performed on parental peripheral blood using the same platform as the proband. Neither parent was noted to carry the duplication. In addition, routine chromosome analysis was performed, and neither parent possessed a detectable balanced rearrangement involving chromosome 8 in lymphocytes. Thus, the 8p23.1 duplication identified in the fetus was presumed to be de novo, though parental gonadal mosaicism and small insertional balanced translocation could not be ruled out.

The patient was counseled at the time of the amniocentesis procedure regarding the poor fetal prognosis given the severity of the structural anomalies, specifically that these would likely be incompatible with survival. The patient elected to continue the pregnancy. Subsequent ultrasound at 34 weeks' gestation confirmed the presence of the omphalocele, measuring 5.7 cm in diameter and containing both the liver and stomach. The encephalocele also persisted, and a cyst measuring 3.4 cm in diameter was visualized in the gray matter within the defect. The fetus was noted to be growth restricted with an estimated fetal weight of 1,769 grams (3^rd^ percentile by Hadlock formula) with normal amniotic fluid volume. Note that, an amniotic band was newly detected. Due to the 8p23.1 duplication, a fetal echocardiogram was obtained and demonstrated normal four-chambered cardiac anatomy with normal systolic function and no obvious valve stenosis or regurgitation.

The patient elected comfort care during delivery and the postnatal period, with no electronic fetal monitoring during labor. Cesarean section was reserved for maternal indications only. The patient experienced preterm premature rupture of membranes at 34 weeks 2 days' gestation. Her labor was induced with oxytocin, and she underwent vaginal delivery approximately 14 hours later. Palliative care was initiated by neonatology, and the neonate died at 1.5 hours of life. The omphalocele and encephalocele were reportedly well demonstrated at delivery.

## 3. Discussion

The patient described here showed atypical features of 8p23.1 DS. Previous cases were notable for developmental delay (>90%) and mild dysmorphic features, as well as congenital heart disease (CHD) [1–5]. At present, the clinical significance of duplication of 8p23.1 remains unclear, as several parents possessing the same duplication have been reported as phenotypically normal when compared to their affected offspring. However, equal reports of individuals have been described containing mild phenotypic findings and profound pathogenic variants. Here, we report an additional 8p23.1 duplication, confirmed by amniocentesis, which was incompatible with life due to the severity of congenital anomalies. Our patient may represent a severe form of the disease.

Notably, despite the 8p23.1 duplication containing the *GATA4* and *SOX7* genes, no CHD was evident on fetal echocardiography.*GATA4* is a critical transcription factor for proper mammalian cardiac formation and development and is expressed in both fetal and adult heart tissue. Several case reports suggest *GATA4* is associated with several types of CHD, particularly conotruncal and septal defects [1, 3]. One hypothesis regarding this finding is that the phenotype is a result of the influence by several miRNA which can regulate and influence gene expression. Barber and colleagues have studied this extensively and suggest the interaction of protein complexes amongst these genes may be responsible for phenotypic variability [3]. Note that, the ClinGen dosage sensitivity working group has not found any evidence for triplosensitivity of *GATA4*, nor *CTSB* or *MFHAS1*, these being the only three genes in our patient's duplicated region to be reviewed as of the time of publication [6].

None of the identified genes in region 8p23.1 have been independently associated with omphalocele or encephalocele. Thus, the anatomical abnormalities in our case may represent incidental findings as opposed to a true association with 8p23.1 DS. The concomitant presence of omphalocele and neural tube defects has been described in cases of Trisomy 18. Attempts to identify specific genes implicated in both of these anomalies have been investigated, though few have been definitively associated; though of note, when the *SCRIB* gene on the long arm of chromosome 8 is duplicated in mouse models, craniorachischisis is observed [7]. Further research may elucidate a role for this gene in the phenotype present in this patient associated with 8p23.1 duplication syndrome.

Clinically, due to the variable penetrance and expressivity of the associated genes, clinicians can provide only limited information regarding prognosis to parents in cases of prenatally-diagnosed chromosomal copy number variants. Arguably, given the role of *GATA4* and the associated morbidity of CHD, it is reasonable to offer patients with a prenatal diagnosis of 8p23.1 DS a fetal echocardiogram. However, the degree of developmental delay remains unpredictable. Our case demonstrates severe congenital anomalies which have never been reported in association with 8p23.1. It is possible that this case represents expanded phenotypic variability of the 8p23.1 DS, which may play a role when counseling prospective parents faced with this rare diagnosis.

## Data Availability

The genomic microarray data used to support the findings of this study are included within the article. The ultrasound examination data used to support the findings of this study are restricted by the Detroit Medical Center Institutional Review Board in order to protect patient privacy.
